# Development, implementation and usefulness of an intervention to support psychological resilience during the COVID-19 pandemic: a study from a Swedish hospital based on interviews, documents and a survey

**DOI:** 10.1136/bmjopen-2023-081095

**Published:** 2024-08-07

**Authors:** Mariel Taxén, Sara Tolf, Sophia Appelbom, Aleksandra Sjöström-Bujacz, Ninveh Baresso, Karin Herber, Annika Johansson, My Keita, Lotta Ramstedt, Anna Wik Bringby, Terese Stenfors, Pamela Mazzocato

**Affiliations:** 1 Department of Learning, Informatics, Management and Ethics, Medical Management Centre, Karolinska Institutet, Stockholm, Sweden, Karolinska Institutet, Stockholm, Sweden; 2 Södertälje Hospital, Södertälje, Sweden

**Keywords:** organisational development, change management, qualitative research, COVID-19, stress, psychological

## Abstract

**Abstract:**

**Objectives:**

A range of interventions to support psychological resilience among healthcare workers were provided in hospitals during the COVID-19 pandemic. Most research has focused on the content and experience of these interventions, but less is known about their implementation. The aim of this study was to increase understanding of the development, implementation and perceived usefulness of an intervention to support psychological resilience among healthcare workers at a Swedish hospital during the pandemic.

**Design:**

This study employs interviews and documents to explore the development and implementation of support activities and a survey to assess the usefulness of the activities (on a scale from 1 to 5). Qualitative content analysis was used to analyse the interview data and documents. Descriptive statistics were used to analyse the survey data.

**Settings:**

A tertiary hospital in the outskirts of Region Stockholm.

**Participants:**

Eight individual interviews were conducted with actors involved in developing the intervention. 286 healthcare workers answered the survey.

**Results:**

Support activities were developed and implemented by an internal multidisciplinary group who continuously identified and adapted activities to the needs of staff. The strategy of involving existing resources to jointly develop and continuously adapt staff support activities was beneficial for the implementation and longevity of the intervention. Scheduling difficulties were one of the challenges. The mean ratings of the activities ranged from 2.27 for one-on-one counselling to 3.25 for physical activity. Licensed practical nurses generally valued the activities higher than other professional groups.

**Conclusions:**

The provision of activities to support psychological resilience in a crisis is facilitated by the ability of an organisation to use current resources in the face of a crisis, which is a sign of organisational resilience. Leaders who act quickly and create the conditions to test and learn under uncertainty can contribute to developing effective responses to a crisis.

**STRENGTHS AND LIMITATIONS OF THIS STUDY**

- This study employs multiple data including interviews, documents and surveys.
- The qualitative content analysis was primarily carried out by an experienced researcher in Medical Management together with a registered nurse with working experience from the pandemic.
- The survey was designed for a larger study that investigated the psychological well-being among healthcare workers in multiple settings, and it did not evaluate all the specific activities provided to support psychological resilience in the studied hospital.
- Interviews were conducted in retrospect, increasing the risk of recall bias.

## Introduction

 The COVID-19 pandemic made stress and burnout among healthcare workers^1^,^3^ a major issue of concern in many settings.^4 5^ Frontline workers experienced challenges concerning fear of contracting or transmitting a new and potentially deadly virus to family members, lack of personal protective equipment, lack of guidelines and information and poor preparation of working in a crisis.^6^ Several studies confirmed that the prevalence of stress, anxiety and depression was high among staff caring for COVID-19 patients.^7^,^9^

The necessity of promoting mental well-being and psychological resilience of healthcare workers became even more urgent during the pandemic.^8 10^ Psychological resilience has been defined as a protective defence mechanism against developing ill mental health and refers to the ability to ‘maintain relatively stable and healthy levels of psychological and physical functioning’ under stressful or threatening conditions.^11^

Workplace interventions have been described to help increase the psychological resilience of healthcare workers and reduce the risk of long-term mental health problems.^12^ A range of different psychological support interventions were implemented in hospital settings around the world during the COVID-19 pandemic,^13 14^ but what promotes resilience in healthcare workers is still not fully understood.^10^ Among the described initiatives are interventions expanding services for basic needs, interventions to support preparedness, increased access to mental health treatment, physical activity, music therapy, and mobile apps to support mental health.^14 15^

The content of psychological support interventions, as well as the experience of providing them, have been described in scientific studies,^16 17^ however less focus has been directed towards the implementation of these interventions.^18^ The unpredictable and chaotic nature of the pandemic require organisational and leadership responses that differ from those needed in a more stable and ordered environment.^19^,^22^ Organisational capabilities to quickly but wisely reconfigure in chaotic environments and crisis management strategies that respond to unexpected events are key for an organisation when confronting unpredictable and sudden change as the pandemic initially represented.^19^,^21^ Empirical studies on implementing staff support during the pandemic^18^ as well as theories of change in healthcare^23^ indicate that interventions that are flexible, adaptable and tailored to meet local needs are more likely to be successfully implemented.

The aim of this study is to increase understanding of the development and implementation of an intervention to support psychological resilience at a Swedish public hospital during the pandemic. The three research questions are: How were activities to support psychological resilience among healthcare workers developed and adapted in a Swedish hospital? What facilitated and hindered the implementation of the support intervention? and How did participants of the support activities perceive their usefulness?

## Methods

### Study design

This study employs multiple methods including interviews, documents and surveys to explore development and implementation of activities to support psychological resilience during the period March 2020-–April 2022.

### Study setting

The study took place at a public tertiary hospital in the outskirts of Stockholm with approximately 1050 employees. The hospital is a combined emergency and local hospital, offering planned specialist-care including childbirth, and local care such as geriatric, palliative and advanced home care.

In late February 2020, the Swedish Public Health Authority assessed the risk of spreading COVID-19 in Sweden as very low. However, the first death from COVID-19 was reported on March 11, and in the middle of April 2020, the daily deaths from COVID-19 and the number of daily new admissions to the intensive care units (ICUs) peaked.^24^ In the hospital studied in this research, the ICU scaled up the number of beds from 6 to 70, and staff were transferred from their regular work units to the ICU. Most of them had no previous experience of providing intensive care and some objected to work with COVID-19 patients. Guidelines for how to use personal protective equipment and treat patients were uncertain and often changed. Each emergency hospital in Region Stockholm had a crisis support group that was affiliated to the regional Crisis and Disaster Psychology Unit. The Unit lead and organised crisis support to patients, relatives and to some extent healthcare workers.^25^

In this context, efforts to support psychological resilience among healthcare workers were launched in March 2020 by a multidisciplinary support group that also included members from the crisis unit. Initially the support was intended for healthcare workers treating COVID-19 patients, but the mission was soon expanded to include all hospital staff.

### Data collection

Data were collected through semistructured interviews, documents and surveys.

Semistructured interviews were conducted between August and December in 2022. A semistructured interview guide (online supplemental appendix 1) with open-ended questions was developed to explore the psychological resilience support activities, their content and how and why they were developed and implemented.

Interviewees were purposively selected among the 20 members of the support group as they were deemed to have relevant knowledge on the development and implementation of staff supporting activities. Out of these, eight people were selected to represent the variety of different professions in the group, as well as the different activities that were developed to support staff. All the eight people agreed to participate in the study. Seven participants were women, and one man. The selection was deemed to provide satisfactory information power^26^ since the participants had specific knowledge about the support activities, and the sample was deemed sufficient to draw conclusions on this exploratory study with focused research questions.

All interviews were audiorecorded and conducted through video calls by ST who interviewed from her office, and participants took part from their workplace at the hospital. The participants were informed about the objective of study. Interviews lasted for about 1 hour each.

Documents including logbooks, meeting protocols, presentations and reports concerning the activities were collected through the members of the support group for the period March 2020 to the spring of 2022.

The survey was part of a larger study, investigating the psychological well-being among healthcare workers over time during the pandemic.^27^ The survey was sent to all hospital staff based on convenience sampling. A total of 1700 people received the survey in June 2020 based on a list provided by the HR department, but only 1400 were estimated to still work at the hospital when the study was conducted. Three follow-ups were disseminated in September 2020, February 2021 and June 2021. In total, 462 workers at the hospital participated in the survey, representing around 40% of the workforce.^27^ For the present analysis, data on the perception of support activities measured at the second follow-up in February 2021 (n=286) were used. Participants who indicated that they had been offered support activities during the pandemic were asked to rate the usefulness of each activity on a scale from 1=Not at all appreciated to 5=Very much appreciated. The types of rated support activities were educational support on stress response, counselling conversations in groups, one-on-one counselling and physical activity.

### Data analysis

Interviews were transcribed and analysed using qualitative inductive content analysis, which was deemed suitable given the exploratory nature of the study and the novelty of the phenomenon.^28 29^ Data analysis was primarily conducted by ST, an experienced researcher with a PhD in Medical Management and with extensive experience in qualitative methods, and MT, an registred nurse (RN) with work experience from the COVID-19 pandemic and pursuing a master’s degree in health economics, policy and management. Both are female and with no previous links to the hospital or the study participants. They identified meaning units, shortened them into condensed meaning units, developed codes and sorted them into categories and subcategories based on their manifest meaning. The sorting of codes into categories and subcategories was first done individually and in silence by the two researchers. When all codes were sorted, they discussed until they reached an agreement on the content and labels of categories and subcategories. The raw data wear reviewed repeatedly to assure the accuracy of the codes, categories and subcategories. Finally, themes were formulated to capture the answer to the two qualitative research questions. QRS NVivo V.12 Plus software was used for coding the data and MIRO online whiteboard for visual collaboration was used for sorting data into code-groups, categories and themes. All interview participants and the coauthors were invited to a validation meeting during which the results of the analysis were presented and discussed. Moreover, all participants were offered to read the results, and some of them provided feedback.

Documents were read through, and data were extracted concerning the content of the activities and the time when they were delivered. This data were summarised in tables and a timeline.

Descriptive statistics were used to analyse survey data. The statistical software Jamovi V.2.3.21.0 was used to visualise the quantitative results.

Contents from the multiple data sources, particularly documents and interviews, were compared with each other to assess consistency of the results.

### Patient and public involvement

Members of the multidisciplinary support group who developed and carried out the activities to support psychological resilience in the studied setting are coauthors of this research paper and have contributed to the research as outlined in the ‘Authors’ Contribution’ section.

## Results

In this section, the findings of the qualitative analysis are presented first, followed by quantitative results on the perceived usefulness of the interventions.

### Content, development and implementation of the supporting activities

The content of the different activities identified, their purpose, the intended target group, as well as the number of staff who participated in the activities are displayed in table 1. In total, based on logbooks, approximately 2283 staff members participated over time in the activities provided in 295 occasions (ie, the same person was counted for each time he/she attended an activity).

**Table 1:** Description of the support activities

Support activity	Short description	Target group	Number of times provided	Number of participants*
Counselling conversations in groups	Counselling conversations of a relieving character. Eventually the conversations changed towards a reflective approach	Initially frontline workers who worked with COVID-19 patients. Eventually also healthcare workers in other units	201	1460
One-on-one counselling conversations	Individual conversations with a member from the staff supporting group	Healthcare workers and managers	116	116
Reflection and education for managers	Reflection together with other managers. Drop-in sessions and eventually mandatory gatherings. Lectures on crisis management	Managers	17	123
Managerial mingle	Outdoor meetings to socialise under relaxed circumstances	Mangers	2	18
Educational support on stress response	Lecture on the body’s reaction to stress and the brain’s motivational system	Healthcare workers and managers	21	336
Mandala	Colouring books and pens for a moment of relaxation	Distributed in four hospital wards, for healthcare workers	NA	NA
Brochure on recovery	Information about recovery during vacation. Contact information for crisis support	Healthcare workers	NA	NA
Reflection material for managers	Cards with pictures aiming to facilitate reflective conversations with healthcare workers	Managers	NA	NA
Physical activity				
Yoga and mindfulness	Medi-yoga and chair yoga	Healthcare workers	12	74
Tactile massage	Massage provided by two people from the support group	Healthcare workers in the intensive care unit	8	28
Relaxation and conversations	Relaxation in groups often in combination with light-hearted conversations	Healthcare workers	18	128

* Represents the total number of participants, not the unique number of participants.**Represents the number of participants from one of the two occasions. Applicable

† Represents the number of participants from one of the two occasions.

**Figure 1:**
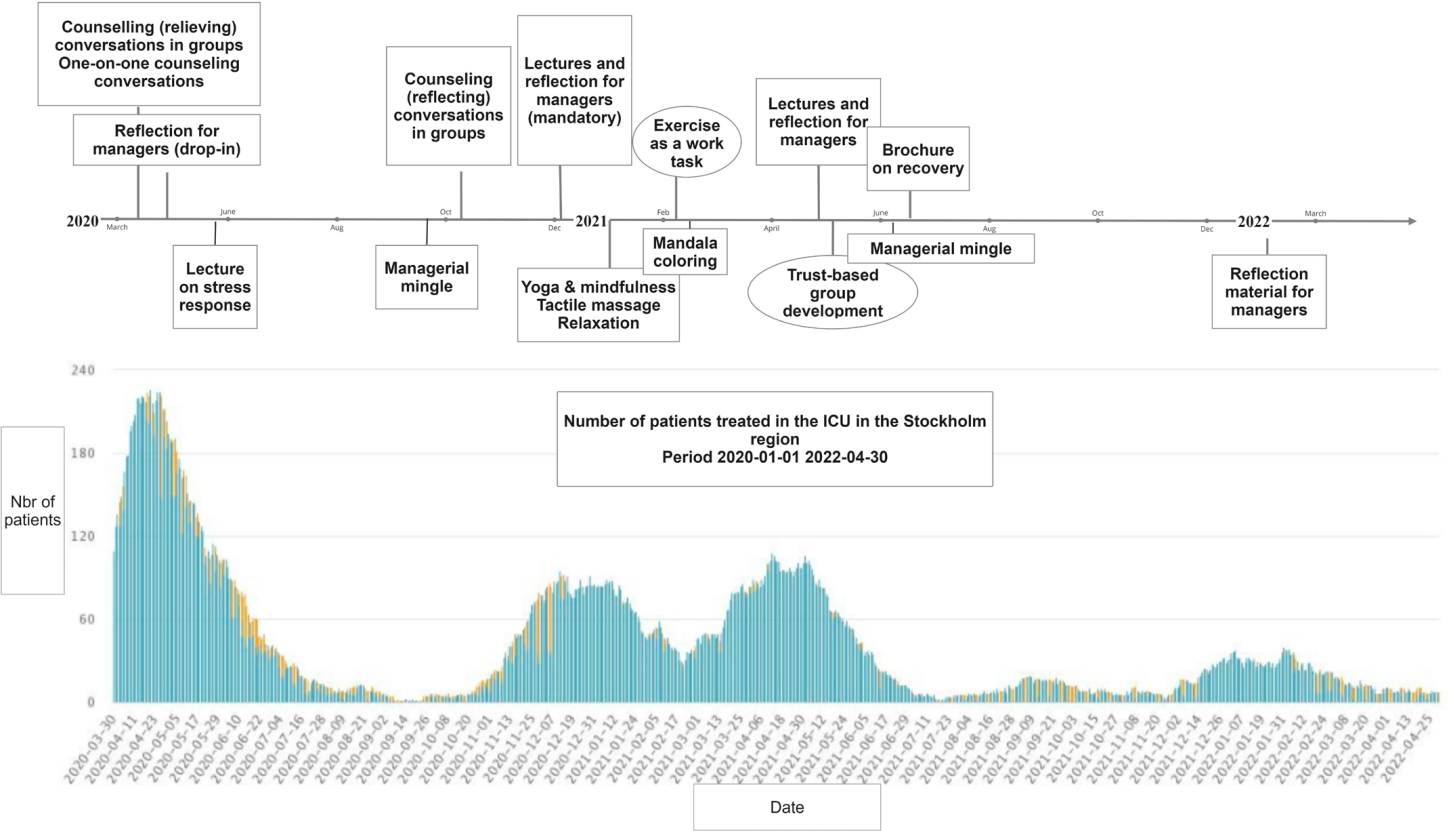
Timeline of support activities for psychological resilience during different phases of the pandemic. Graph displaying the pandemic waves is retrieved from The Swedish Intensive Care Registry (SIR).^30^ ICU, intensive care unit.

In figure 1, the activities are presented in a timeline for the period March 2020–April 2022 in relation to the pandemic waves in Sweden.^30^

The qualitative content analysis also resulted in the identification of two themes on the development and adaption of support activities for psychological resilience, and facilitators and hinders for implementing the support intervention.

### Theme 1: act quickly, use internal resources and enable learning and adaptation

Theme 1 includes categories and subcategories that are displayed in table 2 and described below.

**Table 2:** Act quickly, use internal resources and enable learning and adaptation

Categories	Subcategories
Support activities were initiated by the Crisis and Disaster Psychology Unit, encouraged by the hospital management and formalised throughout the organisation	The initiative came from the Crisis and Psychology Unit Recognition for the need of psychological support Management mandates the continuation of psychological support
The support group was formed based on an inventory of internal diverse competence and with the support of an external consultant	Responsibility for the intervention Consulting assistance in the beginning Internal competence joined voluntarily Different professions and hospital specific programmes
Cooperation across borders to identify and adapt to current needs	Working across professional boundaries Different ways of identifying needs Adaption of activities
The longevity of activities and a call for a sustainable working life	Contributing to organisational changes The longevity of interventions

#### Support activities were initiated by the Crisis and Disaster Psychology Unit, encouraged by the hospital management and formalised throughout the organisation

In early 2020 there was a dialogue between physicians in the ICU and a member of the hospital’s counselling and psychology unit regarding signals from the strained healthcare situation in Italy, due to the COVID-19 outbreak. The member of the counselling and psychology unit, who was also part of the Crisis and Disaster Psychology Unit, proposed to start providing debriefing sessions with staff in the ICU.

…we had a lot of contact with one of the (ICU) chief physicians at the time who also warned us about the situation in Italy, that the staff were tired. The staff couldn't cope, they were crying, they were having a really tough time and then they said - how should we think here at the hospital? Interview participant 3

In March 2020, the CEO of the hospital formalised the mission to support psychological resilience by mandating the development and dissemination of support activities across the hospital.

#### The support group was formed based on an inventory of internal competence and with the support of an external consultant

The head of the Research & Development, Education and Innovation department, together with a staff member from the Human Resource (HR) department got the responsibility to lead and organise the support intervention. They started off together with the colleagues who were already providing debriefing sessions in the ICU.

Well, when the CEO and the management team came and said we were going to do this, we had already started. We had been at it for 14 days, three weeks or so. Interview participant 5

The start-up and organisation of the support group was initially assisted by an external consultant with extensive experience of working with staff support in crisis situations and training in counselling skills. The support group was expanded through an inventory of staff at the hospital with competences relevant for the mission of the group. Some were reached via emailing unit managers, others joined voluntarily.

I think there was a lot of commitment and willingness to contribute…we just picked those who actually raised their hands. When we did a reconciliation of the support group, there were about 20 people who had been part of this group. Interview participant 2

At maximum, the support group was composed by 20 people with different professional backgrounds, that is, counsellors, psychologists, nurses, managers, hospital’s chaplains, a wellness consultant, representatives from HR and Research & Development, Education and Innovation. Two of the participants were also members of the hospital management team.

#### Cooperation across borders to identify and adapt to current needs

The support group cooperated across professional boarders and was granted the time needed to work in the group. Cooperation was described in terms of learning from each other, taking advantage of everyone’s input, helping each other, brainstorming together and learning along the way. There was a positive work climate during the meetings which allowed participants to contribute with their unique competence.

There were no experts in the hospital who knew: This is how to do it. Instead, we went in with what we had and did something together. Interview participant 5

Initially the group met once or twice a week to coordinate and plan the support activities. Typically, a request for support from the floor could be addressed within the same day or the day after. When the demand for support was high, the group had access to external consultants. In addition to the support offered by the support group, staff working at the hospital also had the option of choosing external crisis support around the clock, offered by the occupational health service.

The support group aimed to constantly tune into the changing needs of staff based on feedback and input they received from the staff and managers during the support activities, at meetings, through spontaneous encounters, through e-mail, and based on the results from the survey investigating mental well-being.

Support activities were adapted to the different needs arising during the different phases of the pandemic, and to the diverse needs of different staff groups. For instance, when the number of participants in the counselling group conversations decreased, the group reasoned that something else was needed. The character of group conversations was therefore changed from the initial relieving approach to a more reflective approach. This continual adaptation process resulted in the group experimenting with different activities, such as mandalas, yoga and wellness challenges (table 1).

Yes, I was about to say: It was changing all the time!…There were fairly short periods when we had a situation where we knew what we were doing, and we knew what we would do next week and so on. But it was always an adaptation to the needs that existed. Interview participant 5

Observations made by the support group on staff’s traumatic experience of being transferred from their original workplaces during the first wave helped to ensure that staffing could be resolved on a voluntary basis rather than on a mandatory basis during the second wave.

#### The longevity of activities and a call for a sustainable working life

The support group officially operated between March 2020 and April 2022. Two projects that were initiated by the group were continued and permanented at the hospital based on a decision of the CEO and the top management. The first was ‘training as a work task’, to implement physical activity as part of daily work. During the fall 2020, the wellness consultant recruited healthcare workers to be trained as leaders of short exercise sessions, to create a network of health influencers in the hospital. The second activity was a ‘trust-based group development programme’ that focused on how to build trust in teams.

### Theme 2: commitment, closeness and preparedness facilitated implementation, while practical challenges were faced

Theme 2 includes categories and sub-categories that are displayed in table 3 and described below.

**Table 3:** Commitment, closeness and preparedness facilitated implementation, while practical challenges were faced

Categories	Subcategories
Preparedness in a small hospital characterised by good relations and organisational knowledge*	Preparedness through training in crisis support in the Crisis and Disaster Psychology Unit organisation Good relations and organisational knowledge in a small hospital Experience of working with improvements
A committed and involved management that enabled internal resources to work in a flexible, rapid and supportive way*	An unpretentious and supportive management who managed without hindering Rapid decision-making and flexibility by using internal resources in combination with external Participation in the support group was supportive
Difficulties related to scheduling and reaching out with support†	Scheduling difficulties of meeting demand at the same time all over the hospital and extending activities beyond working hours Reaching out with information about support and reaching physicians and night shift workers
The challenge of harmonising and including different perspectives†	Differences in the approach to the provision of support Perceived prestige linked to level of education and type of support offered

* Facilitators.

† Hinders.

#### Preparedness in a small hospital characterised by good relations and organisational knowledge

In early 2020, several members of the Crisis and Disaster Psychology Unit management team had participated in a regional training called ‘staff supporting interventions in case of extraordinary events’. Although some of the Crisis and Disaster Psychology Unit members had previous experience of providing support, the training was described to strengthen their confidence and preparedness to be able to support staff when the pandemic hit Sweden.

Several of us had a sense of security that yes, we can handle going into this! I would probably have felt much more insecure to sit down and have a debriefing with such a large group who were working with something that was traumatizing for them if I hadn't had these days and sort of reasoned about it. Interview participant 5

Good relations, knowledge of the hospital organisation, a culture that value staff suggestions for improvement and easy access to colleagues were all deemed facilitating factors.

…we knew exactly how things worked in different departments and had local knowledge…we could fine-tune! And we could adapt who in the support group was suitable for certain units, and we could fine-tune because we had local knowledge. Interview participant 7

#### A committed and involved management that enabled internal resources to work in a flexible, rapid and supportive way

The hospital management and the leaders of the support group were described as unpretentious and supportive.

But as I said, I would say that what made this work very well was that X (member of the management) did not come in with an idea of how this should be done, but the attitude as I perceived it, was a lot like: OK none of us have really done anything like this before. How can we make sure that this works as well as possible? And then we worked it out together. Interview participant 5

The support group was quick to start providing support, which was facilitated by rapid decision-making in the organisation, and the use of internal resources despite some initial support from an external consultant in the start-up phase.

…we were the hospital, I think it’s quite unique, that started quite quickly with internal resources. And offering crisis support interventions, some hospitals I think only took help from external ones. Interview participant 3

The majority of the participants expressed that participation in the group had been a positive experience. Many described that the work atmosphere was free from hierarchy, supportive and with everyone seen as equal. This allowed for brainstorming and testing new ideas. There was also a feeling of making a difference. The group focused on learning along the way which was considered a key factor for the implementation.

I've had it well arranged for me, so to speak, because I've had this group. Incredibly good people, wise and reflective and all that entails. So it has been a benefit for me, absolutely. Interview participant 6

#### Difficulties related to scheduling and reaching out

To schedule the support activities was one of the most commonly described difficulties. Most units wanted support at the same time every day, usually in the shift between morning and afternoon staff, which would have required more people in the support group. Another difficulty was motivating staff to participate in the support activities as the activities sometimes were carried out beyond working hours.

Physicians were difficult to engage in the activities. It was expressed that they might want to manage on their own, and one participant noticed that nurses, in comparison, were more open to be vulnerable. Night workers were also challenging to reach, although some attempts were made.

…we worked mostly on the wards with assistant nurses and nurses but the groups of doctors are organized in such way so it was more difficult to reach them, and it was very difficult …I think there was a bit of a bad feeling if you have to go and talk about this kind of thing. Interview participant 1

Many respondents pointed out the importance of being seen by the hospital management. However, one participant mentioned that the ICU received a lot of attention while other units struggled behind the scenes.

#### The challenge of harmonising and including different perspectives

One participant described that the different professional backgrounds among the participants in the support group resulted in that the approach to delivering support differed depending on who was providing it. The respect shown to each other’s professions was thereby described as an obstacle as it became difficult to streamline the psychological support.

I feel that it was a bit too therapeutic, as I said, because we are not supposed to treat, we are supposed to remedy the first problems. Interview participant 1

Although most of the participants in the support group described the atmosphere as unpretentious, there were also opinions to the contrary. One participant expressed a sense of prestige linked to educational level and the type of support one offered.

### Perceived usefulness of the support activities

Ratings on the perceived usefulness of the support activities are presented in table 4. Among the survey participants, 212 rated the usefulness of at least one of the support activities. Out of these 81 (47%) were frontline workers, which meant daily or several times a week care of COVID-19 patients. The most common occupational groups were nurses (n=70, 34%) and other occupations (n=69, 33%), followed by licensed practical nurses (LPNs, n=45, 22%) and physicians (n=24, 12%).

**Table 4:** Usefulness ratings of support activities broken down by occupational group

Activity	LPN	Nurses/midwives	Physicians	Other occupations	Total
	nM (SD)	nM (SD)	nM (SD)	nM (SD)	nM (SD)
Educational support on stress response	n=363.36 (1.02)	n=582.88 (1.26)	n=202.40 (1.35)	n=592.90 (1.11)	n=1762.94 (1.20)
Counselling conversations in groups	n=263.58 (1.21)	n=283.04 (1.26)	n=112.45 (1.37)	n=373.14 (1.00)	n=1302.68 (1.31)
One-on-one counselling conversations	n=242.83 (1.37)	n=362.36 (1.46)	n=131.38 (0.65)	n=382.11 (1.27)	n=1122.27 (1.35)
Physical activities	n=193.37 (1.42)	n=283.18 (1.16)	n=52.00 (1.41)	n=303.40 (1.45)	n=833.25 (1.37)

Activities were rated on a scale from 1=not at all appreciated, 2=a little appreciated, 3= to some extent appreciated, 4=much appreciated to 5=very much appreciated.

LPNslicensed practical nurses

The mean ratings of the activities ranged from 2.27 for one-on-one counselling to 3.25 for physical activity. The occupational groups differed in their ratings. Physicians gave the lowest usefulness ratings, ranging from an average of 1.38 (one-on-one counselling) to 2.45 (group counselling). For most activities, LPNs rated the perceived value of the support higher than the other occupations. Their ratings ranged from an average of 2.83 (one-on-one counselling) to 3.37 (group counselling). However, the other occupations group gave a slightly higher average rating on physical activity (3.40). Table 4 presents perceived usefulness ratings on each support activity across all occupational groups.

## Discussion

The findings of this study suggest that the managerial strategy of involving internal resources of the hospital to jointly develop and continuously adapt support activities was beneficial for the implementation and longevity of a psychological resilience support intervention.

In this case, the hospital’s CEO *acted* quickly by formalising the mission and appointing responsibility to support the psychological resilience among healthcare workers. This leadership approach is consistent with current understanding of leadership practices in chaotic situations such as the rapid spread of an unknown virus, a rare event with serious consequences for physical and emotional well-being.^31^ This is for instance exemplified in the Cynefin framework^21 32^ which suggests that in chaotic situations there is a need to *act* to stabilise the situation and create the order needed so that a response can be developed. While the CEO and the hospital management acted promptly, participants described how the management did not command what to do, but rather encouraged *probing* different activities and continuously *sensing* the needs among the workforce. This resulted in a multidisciplinary group that worked together to find solutions. The group collaborated openly and ideas on how to support staff were tested, evaluated and adjusted by the support group. These findings imply that management’s response in chaotic contexts is not only to *act*, but once some form of stability has been established, also to enable learning. In this way patterns emerged, a process described in the Cynefin framework.^21^

The ability to develop and implement staff support in a timely manner was associated with an organisational preparedness (eg, staff with formal training in crisis and disaster management), a work climate characterised by good relations and quick decision-making. These factors contributed to mobilising internal resources and to adapt the activities to the changing needs of the workforce. This approach also allowed the intervention to continue over a 2-year period. Moreover, the lessons learnt from the support activities were incorporated into activities that were continued after the pandemic, such as a new educational programme to foster trust and psychological safety^33^ in work groups. Another study has described efforts to provide psychological support provided by external psychologists.^34^ While positive results were reported in terms of rapid provision of support, and adapting content of support to current needs, the long-term sustainability of these efforts seemed to be one of the major challenges.

The ability of an organisation to use current resources in the face of a crisis is a capability that has been associated as organisational resilience, where using existing resources, redesigning tasks and paying attention to team emotions have been described as a method to create resilient teams and organisations.^35^ Organisational resilience has further been conceptualised as a process including the three capabilities *anticipation*, *coping* and *adaptation*, enabling the organisation to respond in different phases of a crisis.^19^ This case illustrates the example of an organisation that was capable of identifying the critical developments of the pandemic and prepared to support staff (*anticipation*). A fast response through coordinated efforts enabled to develop and implement support activities (*cope*). Lessons from the crisis were incorporated into new practices after the pandemic (*adaptation*).^21^

Despite the success in developing and implementing support activities, the survey data show a mild appreciation from participants of the activities. The fact that half of the participants did not work in the frontline and the timing of the survey (between the second and third wave of the pandemic) may explain why the activities were not rated higher. It is likely that the support had been perceived as more useful if measured during the first pandemic wave when stressors related to the rapid increase of patients, traumatic events and fear of personal safety were more salient.^36 37^ However, the results showed that LPNs, who work closest to the patients, generally valued the activities higher than other professional groups. During the COVID-19 pandemic, direct patient care has been linked to increased demands, such as moral stress.^38^ The need of support may therefore have been stronger among the LPNs. Participants with 'other occupations' rated physical activity highest of all, which seems reasonable for occupational groups with more sedentary jobs. Perhaps the activities themselves were not as important as the fact that the organisation was willing to support and invest resources in the well-being of its staff. Healthcare workers want unambiguous assurance that their organisation will support them, and by asking what they want, and doing the utmost to meet their needs is appreciated, even if not all requests can be met.^39^

### Methodological considerations

The main strength of the study was that it captured a real-world case of a hospital-based initiative to support psychological resilience among healthcare workers. The case is unique because of the breadth and longevity of the activities provided. As with qualitative studies in general, transferability is determined by the description of the context and of the participants provided in this study.

Several strategies were employed to ensure trustworthiness. In the preparation phase, three sources of data were selected that together provided reach and complementary data on the activities developed, the implementation timeline, the experience of staff involved in the support activities, and the perceptions of staff.

Another strength of the study was the diverse background of the researchers, including several experienced researchers in the field of healthcare organisation and management, and in organisational psychology; an RN with experience of clinical work during the COVID-19 pandemic; several healthcare professionals with experience of clinical work and of delivering supporting activities. The mix of competences contributed to the understanding and interpretation of the phenomenon studied. The risk of bias associated with the fact that several of the authors were involved in the initiative were mitigated by data collection and analysis being primarily conducted but the two authors external to the organisation (ST, MT).

The qualitative analysis process was carried out with great attention to the chosen methodology, was conducted primarily by two researchers and involved repeated discussions to ensure agreement within the research team on the reliability of codes and categories. Results were also subject to informant validation.

One of the limitations of the study was the number of interviewees. While the number of participants was deemed sufficient given the exploratory and focused scope of the study, the inclusion of participants from a larger group, including staff members and managers, may have provided more in-depth understanding of how the support activities were received. However, all interviews provided a rich and detailed picture of the phenomenon, and combined with the extensive document analysis, the analysis of how the intervention developed was consistent. The document analysis was an asset also given that the interviews were conducted in retrospect, and time details were difficult for some interviewees to recall.

Another limitation was that the survey was developed for a larger study in multiple settings, thus not all activities offered by the support group were evaluated.

## Conclusion

This study provides an example of how an intervention to support psychological resilience among healthcare workers during a pandemic can be developed and implemented by using internal resources and allowing for support activities to be iteratively adapted to the contextual needs. Managers who lead in a crisis can create the conditions for psychological resilience by mobilising existing resources, acting quickly and creating the conditions to test and learn under uncertain conditions. These conditions allow a fast response and the capability to incorporate lessons learnt even in the aftermath of the crisis. Further studies could include multiple cases to better understand the conditions for hospitals to support psychological resilience among healthcare workers in a crisis.

## supplementary material

10.1136/bmjopen-2023-081095 online supplemental file 1

## Data Availability

Data are available upon reasonable request.
