# Supplementary material for: Development, implementation and usefulness of an intervention to support psychological resilience during the COVID-19 pandemic: a study from a Swedish hospital based on interviews, documents and a survey
Source: BMJ Open. 2024 Aug 7;14(8):e081095. doi: 10.1136/bmjopen-2023-081095 (PMC11404210; doi:10.1136/bmjopen-2023-081095)
Supplement: online supplemental file 1 [file bmjopen-14-8-s001.pdf]

Interview guide

Study on staff supporting activities during the pandemic.

Introduction

Thank you for taking the time to participate in this interview!

The purpose of this project is to increase understanding of how staff supporting activities during the pandemic can be organized and implemented. The questions will be about why you chose to implement the activities, what the activities consisted of, how you performed the activities, changes over time and the effects so far. The interview will take about 40-60 minutes. Participation is voluntary. You can choose to withdraw from the interview at any time. All data will be treated confidentially. Participation in the interview is anonymous in that you will not be mentioned by name in any produced document, only our research group at MMC will have access to the interview material.

If you have any questions, you can contact us via email sara.tolf@ki.se

To facilitate the analysis work, the interview will be recorded. Is it OK for you? Then I start the recording. And then I'll repeat the question, is it ok for us to record?

Questions

Introduction

1. Could you briefly describe your background and how long you worked at the hospital? What is your role in the organization and your role in the staff supporting activities during the pandemic?

Program theory

2. Can you describe the background to why you started the activities? Why did you start with the staff supporting activities? What was the situation like? What problem were you trying to solve? When did you start? What has driven you? (
3. When did you start the efforts?
4. Where did the idea come from? On whose initiative was it to begin the staff supporting activities? Were you inspired by anyone? Any literature?
5. What did the working group look like? Who attended? (internal/external?)
6. How have you been working? Did you have a particular working method/model for working with the project group?
7. What kind of leadership has been required to carry out the activities?
8. How was the staff supporting activities financed?

9. Tell us about the staff supporting activities. What activities have you implemented? We take one activity at a time:

   A) What was the purpose of the activity?
      i. What have you done to achieve that purpose?
      ii. How does it achieve the purpose?
      iii. How do you know if the purpose has been achieved? How do you measure?
      iv. What effects have you seen? (How did the staff experience it?) Any
      v. Unexpected effects?
      vi. Have there been any changes in the meantime?

   B) (the questions continue for each activity)

10. What have been the biggest difficulties/obstacles in implementing the activities?
11. If you were to do it all over again, is there anything you would have done differently?
12. What has been the most successful about the way of carrying out the activities?
13. In retrospect, how would you say that working with the staff supporting activities was made easier or more difficult by the fact that you were at this hospita)?
14. How did you change the activities over time and why?
15. How has the context affected your work? Influence from outside?
16. What does the future look like? Do you continue with the activities? Development?

17. Is there anything else you would like to add?

Many thanks for the interview!

sara.tolf@ki.se
