# Supplementary material for: Development, implementation and usefulness of an intervention to support psychological resilience during the COVID-19 pandemic: a study from a Swedish hospital based on interviews, documents and a survey
Source: BMJ Open. 2024 Aug 7;14(8):e081095. doi: 10.1136/bmjopen-2023-081095 (PMC11404210; doi:10.1136/bmjopen-2023-081095)
Supplement: Supplementary file 2 [file bmjopen-2023-081095.reviewer_comments.pdf]

PEER REVIEW HISTORY

BMJ Open publishes all reviews undertaken for accepted manuscripts. Reviewers are asked to complete a checklist review form and are provided with free text boxes to elaborate on their assessment. These free text comments are reproduced below.

ARTICLE DETAILS

**TITLE (PROVISIONAL)**	Development, implementation, and usefulness of an intervention to support psychological resilience during the COVID-19 pandemic: A study from a Swedish hospital based on interviews, documents, and a survey.
**AUTHORS**	Taxén, Mariel; Tolf, Sara; Appelbom, Sophia; Sjöström-Bujacz, Aleksandra; Baresso, Ninveh; Herber, Karin; Johansson, Annika; Keita, My; Ramstedt, Lotta; Bringby, Anna Wik; Stenfors, Terese; Mazzocato, Pamela

VERSION 1 - REVIEW

**REVIEWER NAME**	Farahani, Hojjatollah
**REVIEWER AFFILIATION**	Tarbiat Modares University, Psychology
**REVIEWER CONFLICT OF INTEREST**	There is no conflict of interest.
**DATE REVIEW RETURNED**	08-Jan-2024

**GENERAL COMMENTS**	Many thanks for sharing your manuscript, The following issues need to be considered:<br>1-What is the type of content analysis? I think it is close to latent content analysis.<br>2-What was criterion of saturation as a tool for stopping the sampling?<br>3-How did you name the obtained themes?<br>4-Why did you justify inductive content analysis as a best qualitative methods for answering to the research questions?<br>5-How did you obtain the trustworthiness of the results?<br>6-Please clarify implications of the results.<br>7-How to deal with non-soft themes?

**REVIEWER NAME**	Duan, Xia
**REVIEWER AFFILIATION**	Tongji University School of Medicine, Department of nursing
**REVIEWER CONFLICT OF INTEREST**	I declare that there are no competing interests.
**DATE REVIEW RETURNED**	24-Mar-2024

**GENERAL COMMENTS**	1 The title of the article focuses on supporting psychological resilience, while the article mainly studies the implementation of intervention measures. The title indicates that the article is qualitative research, but it contains quantitative research.<br>2 The summary is structurally confused, and the results and conclusions are not clearly expressed.<br>3 The keywords should refer to the Medical Subject Headings (MeSH).<br>4 Recommendation for reference of Consolidates Criteria for

	Reporting Qualitative Research (COREO) to improve the research team and process response, research design, analysis and results;<br>5 The quantitative survey data were not shown;<br>6 The conclusion does not introduce the limitations of the study.

VERSION 1 – AUTHOR RESPONSE

Reviewer: 1
Dr. Hojjatollah Farahani, Tarbiat Modares University
Comments to the Author:
Dear Authors
Many thanks for sharing your manuscript, The following issues need to be considered:

1-What is the type of content analysis? I think it is close to latent content analysis.

Response: we have clarified in the data analysis section that the content analysis was manifest.

2-What was criterion of saturation as a tool for stopping the sampling?

Response: we have clarified in the data collection section that we used information power, rather than saturation, to inform when to stop sampling.

3-How did you name the obtained themes?

Response: we have revised the name of the themes to better capture who they answer the two qualitative research questions in the study. We have also clarified in the methods how we arrived at the themes.

4-Why did you justify inductive content analysis as a best qualitative methods for answering to the research questions?

Response: we have clarified in the methods section that the choice of inductive content analysis was motivated by the exploratory nature of the study, and the novelty of the phenomenon studied, i.e. the development and implementation of staff supporting activities during the COVID-19 pandemic.

5-How did you obtain the trustworthiness of the results?

Response: we have summarised in the methodological considerations the strategies used to strengthen trustworthiness.

6-Please clarify implications of the results.

Response: We have rephrased the conclusion to clarify the implications of our study.

7-How to deal with non-soft themes?

Response: apologies, but we were not sure how to interpret this comment. However, in the findings section, we have themes, categories, and sub-categories that seek to describe the breadth of the respondents' view. For example, in the category "The challenge of elevating the perspective of all participants in the support group", we captured differences in participants views concerning how they experienced the atmosphere in the support group.

Reviewer: 2
Dr. Xia Duan, Tongji University School of Medicine
Comments to the Author:

1 The title of the article focuses on supporting psychological resilience, while the article mainly studies the implementation of intervention measures. The title indicates that the article is qualitative research, but it contains quantitative research.
Response: thank you for the valuable feedback! We have revised the title to hopefully better capture the content of the article. The new title reads: “Development, implementation, and usefulness of an intervention to support psychological resilience during the COVID-19 pandemic: A study from a Swedish hospital based on interviews, documents, and a survey”

2 The summary is structurally confused, and the results and conclusions are not clearly expressed.

Response: We have revised the abstract to improve the alignment between all its parts.

3 The keywords should refer to the Medical Subject Headings (MeSH).

Response: We have modified the keywords to refer to MeSH

4 Recommendation for reference of Consolidates Criteria for Reporting Qualitative Research (COREO) to improve the research team and process response, research design, analysis and results;

Response: we have uploaded the COREQ checklist and added the necessary information to the main text.

5 The quantitative survey data were not shown;

Response: We were not sure how to interpret this feedback as the survey results were included in Table 4 as well as in the main text of the manuscript. Please let us know if there is anything more that needs to be clarified.

6 The conclusion does not introduce the limitations of the study.

Response: we have included a “methodological considerations” section in the discussion in which we discussed both the strengths and the limitations of the study. We also included suggestions for future studies in the conclusion that could develop more in depth knowledge on how to provide psychological support in a crisis.

Reviewer: 1

Competing interests of Reviewer: There is no conflict of interest

Reviewer: 2
Competing interests of Reviewer: I declare that there are no competing interests.

VERSION 2 – REVIEW

**REVIEWER NAME**	Farahani, Hojjatollah
**REVIEWER AFFILIATION**	Tarbiat Modares University, Psychology
**REVIEWER CONFLICT OF INTEREST**	
**DATE REVIEW RETURNED**	10-May-2024

**GENERAL COMMENTS**	Many thanks for sharing your paper with this journal.The following issues need to be considered:<br>1-Please determine the type of content analysis you have applided. Why did you used that type?<br>2-Please clarify sampling method in the research.

VERSION 2 – AUTHOR RESPONSE

Thank you for your additional feedback, which we have addressed as described below.

1. Please determine the type of content analysis you have applided. Why did you used that type?

Thank you for your feeedback. In the section "Data analysis" we do describe that we used inductive content analysis based on the manifest meaning. We provide two references to the method: Elo et al. 2007, and Graneheim and Lundman 2004. We also motivate the choice of an inductive approach because of the exploratory nature of the study and the novelty of the phenomenon. Is there anything else you would like us to clarify?

2. Please clarify sampling method in the research.

The purposive sampling methods for the interviewes is described in the data collection method to be purposive. We have rephrased the text a bit to try to make it ever more explicit.

For the survey, we have now clarified that we used convinience sampling.
